# Impact of Bicytopenia on Mortality in Hospitalised Patients With Heart Failure

**DOI:** 10.5334/gh.1425

**Published:** 2025-04-30

**Authors:** Toshitaka Okabe, Tadayuki Yakushiji, Daiki Kato, Hirotoshi Sato, Toshihiko Matsuda, Yui Koyanagi, Katsuya Yoshihiro, Takeshi Okura, Yuma Gibo, Yuki Ito, Tatsuki Fujioka, Shigehiro Ishigaki, Shuro Narui, Taro Kimura, Suguru Shimazu, Yuji Oyama, Naoei Isomura, Masahiko Ochiai

**Affiliations:** 1 Division of Cardiology, Showa University Northern Yokohama Hospital, Yokohama, Japan

**Keywords:** blood disorder, all-cause mortality, bicytopenia, heart failure

## Abstract

**Background:**

Limited data are available on bicytopenia (BC) in patients with heart failure (HF).

**Objectives:**

This study evaluated the association between BC and prognosis in patients with HF.

**Methods:**

This retrospective cohort study enrolled consecutive hospitalised patients with HF. We compared all-cause and cardiovascular mortality between those with and without BC. BC was defined as the combination of any two conditions among leukopaenia, thrombocytopaenia, and anaemia. Propensity score matching and a Cox proportional hazards model were applied.

**Results:**

Among 935 hospitalised patients, 103 patients had BC. Patients in the BC group were older (80.0 ± 12.0 vs. 73.4 ± 14.7 years; *P* < 0.0001), including a higher proportion of females (55.3% vs. 41.7%; *P* = 0.009), had a higher prevalence of atrial fibrillation (51.5% vs 41.1%; *P* = 0.047), had a lower baseline estimated glomerular filtration rate (50.8 ± 24.1 vs. 56.2 ± 23.9 mL/min/1.73 m^2^; *P* = 0.03), and had a higher left ventricular ejection fraction (48.1 ± 16.1 vs. 42.4 ± 15.8%; *P* = 0.0008). Propensity score matching with a 1:1 ratio produced 63 matched pairs. All-cause mortality was significantly higher in the BC group than in the non-BC group (log-rank *P* = 0.069 and Wilcoxon *P* = 0.048); however, cardiovascular mortality and hospitalisation for HF showed no significant differences. In the multivariate Cox proportional hazard model, BC was associated with higher all-cause mortality but not with cardiovascular mortality (hazard ratio, 1.983; 95% confidence interval, 1.008–3.898; *P* = 0.047).

**Conclusion:**

BC was associated with all-cause mortality but not with cardiovascular mortality in patients with HF. BC is an important risk factor for all-cause mortality in patients with HF.

## Introduction

Heart failure (HF) is a widespread disease among the ageing population that causes multi-organ impairment through congestion and hypoperfusion (1). Older patients with HF often have multiple comorbidities. Managing comorbidities is crucial in patients with HF, particularly in those with HF preserved ejection fraction (EF), because comorbidities are associated with prognosis in these patients (1). With the increase in the number of older people, the number of peripheral blood cytopenia cases is expected to increase (2). Patients with HF often present with anaemia, with a prevalence ranging from 21% to 43% (3,4). Anaemia is also an independent risk factor for mortality in patients with HF (5,6). Pancytopenia is associated with poor prognosis in patients with HF with preserved EF (7). Additionally, mice with HF demonstrate lower bone marrow cellularity and impaired CD34+ hematopoietic stem cell reconstitution (8). However, few studies have evaluated bicytopenia (BC) (9). BC is one of the forgotten peripheral blood cytopenias; it is not well studied even in the general population. Although the most common aetiology of BC is non-malignant, the association between BC and HF has not been well evaluated (9). We hypothesised that BC might be a prognostic factor in patients with HF. Assuming this hypothesis was valid, intervention for BC might improve the prognosis in patients with HF. Therefore, given the limited data on BC in patients with HF, this study assessed the association between BC and prognosis in patients with HF.

## Methods

We retrospectively enrolled consecutive patients with HF who were admitted to our hospital between January 1, 2010 and December 31, 2020. The final clinical follow-up was completed on August 31, 2023. We analysed the data from September 1 to December 31, 2023. Data on patient history; systolic and diastolic blood pressure; heart rate; echocardiography reports; medication; and laboratory test results on admission, during hospitalisation, and at discharge were obtained. We mainly used the data at discharge for this analysis because evaluating BC in the acute phase is not suitable. The primary endpoint was all-cause mortality. The secondary endpoints were a composite endpoint of cardiovascular mortality and hospitalisation for HF. We compared these endpoints between patients with and without BC and applied a multivariate Cox proportional-hazards model, as well as Kaplan–Meier survival curve analysis, with propensity score matching.

BC is characterised by decreased levels of two of three blood cell types at discharge. Thus, we defined BC as the combination of any two conditions among leukopaenia (white blood cell count <4000/μL), thrombocytopaenia (platelet count <100,000/μL), and anaemia (haemoglobin level <12.0 g/dL in women and <13.0 g/dL in men according to World Health Organization [WHO] criteria) (10). The laboratory data at discharge were used to evaluate the presence of BC.

Two experienced cardiologists independently reviewed patient data, including prescriptions; medical history; echocardiography findings; systolic and diastolic blood pressures; heart rate; and laboratory data on admission, during hospitalisation, and at discharge. Laboratory test results were collected every 1–3 days during hospitalisation. The inclusion criteria were individuals aged ≥20 years diagnosed with acute HF based on the Framingham criteria and biomarkers outlined in the universal definition of HF (11). Briefly, the diagnosis required two major criteria or one major and two minor criteria. The major criteria included orthopnoea or paroxysmal nocturnal dyspnoea, jugular vein distension, rales, cardiomegaly, acute pulmonary oedema, gallop rhythm, increased venous pressure, prolonged circulation time, and hepatojugular reflux. The minor criteria included ankle oedema, night cough, dyspnoea on exertion, hepatomegaly, tachycardia, and weight loss (12). Typically, a plasma B-type natriuretic peptide (BNP) level ≥100 pg/mL at admission was used as an inclusion criterion. The exclusion criteria were pulmonary embolism, acute coronary syndrome, bradycardia necessitating pacemaker implantation, or haemodialysis. Patients who died during HF hospitalisation were also excluded (*n* = 83). Three patients were excluded due to missing data.

This study was conducted in accordance with the Declaration of Helsinki, and the Showa University Research Ethics Review Board approved the protocol. Given the retrospective and observational nature of this study, written informed consent from participants was not required. The information on the study protocol was made publicly available on the hospital’s website, offering patients the opportunity to opt out of the study. This study has been registered within the University Hospital Information Network (UMIN000035989).

## Statistical Analysis

Data were analysed using JMP 17 (SAS Institute, Inc., Cary, NC, USA). Continuous values are presented as the mean ± standard deviation (SD), median (interquartile range, IQR), or total number (percentage). We compared baseline characteristics between the vascular access types using the Mann–Whitney U test for continuous variables and the χ^2^ test for categorical variables. We assessed survival by depicting Kaplan–Meier survival curves and compared the groups with and without BC (the BC and non-BC groups, respectively) before and after propensity score matching using the log-rank test and Wilcoxon test. A two-sided *P*-value of < 0.05 was considered statistically significant. Univariate and multivariate Cox proportional-hazards models were used to estimate the hazard ratios (HRs) and 95% confidence intervals (CIs) for all-cause mortality. The variables fitted in the multivariate analysis included age, sex, history of HF admission, prior myocardial infarction, diabetes mellitus, left ventricular ejection fraction (LVEF), serum creatinine, estimated glomerular filtration rate (eGFR), BNP at discharge, and a prescription of β-blockers, angiotensin-coenzyme inhibitors (ACEI)/angiotensin receptor blockers (ARB) and mineralocorticoid receptor antagonist at discharge. These variables showed *P*-values < 0.10 from univariate analysis or had been demonstrated to be associated with all-cause mortality in patients with HF in previous reports. The propensity score was estimated using a logistic regression model. Independent variables were baseline characteristics found to have statistically significant differences between patients in the BC and non-BC groups, as well as other variables considered to be clinically relevant. The propensity matching included age, sex, atrial fibrillation, history of hospitalisation for HF, haemoglobin levels at discharge, eGFR at discharge, BNP levels at discharge, LVEF, and the prescription of β-blockers and ACEIs/ARBs at discharge. We performed propensity score matching at a 1:1 ratio between the BC and non-BC groups using a nearest-neighbour matching method without replacement and a calliper width of <0.05 × the SD of the logistic score. Variables were compared using the standardised mean difference (SMD), with an SMD <0.20 regarded as well-balanced between the two groups (13). After propensity score matching, the Cox proportional-hazards model was used to estimate the HRs and 95% CIs for all-cause or composite endpoint of cardiovascular mortality and hospitalisation of HF. Regarding hospitalisation for HF, death was treated as a censoring event in the analysis.

## Results

**Figure 1:**
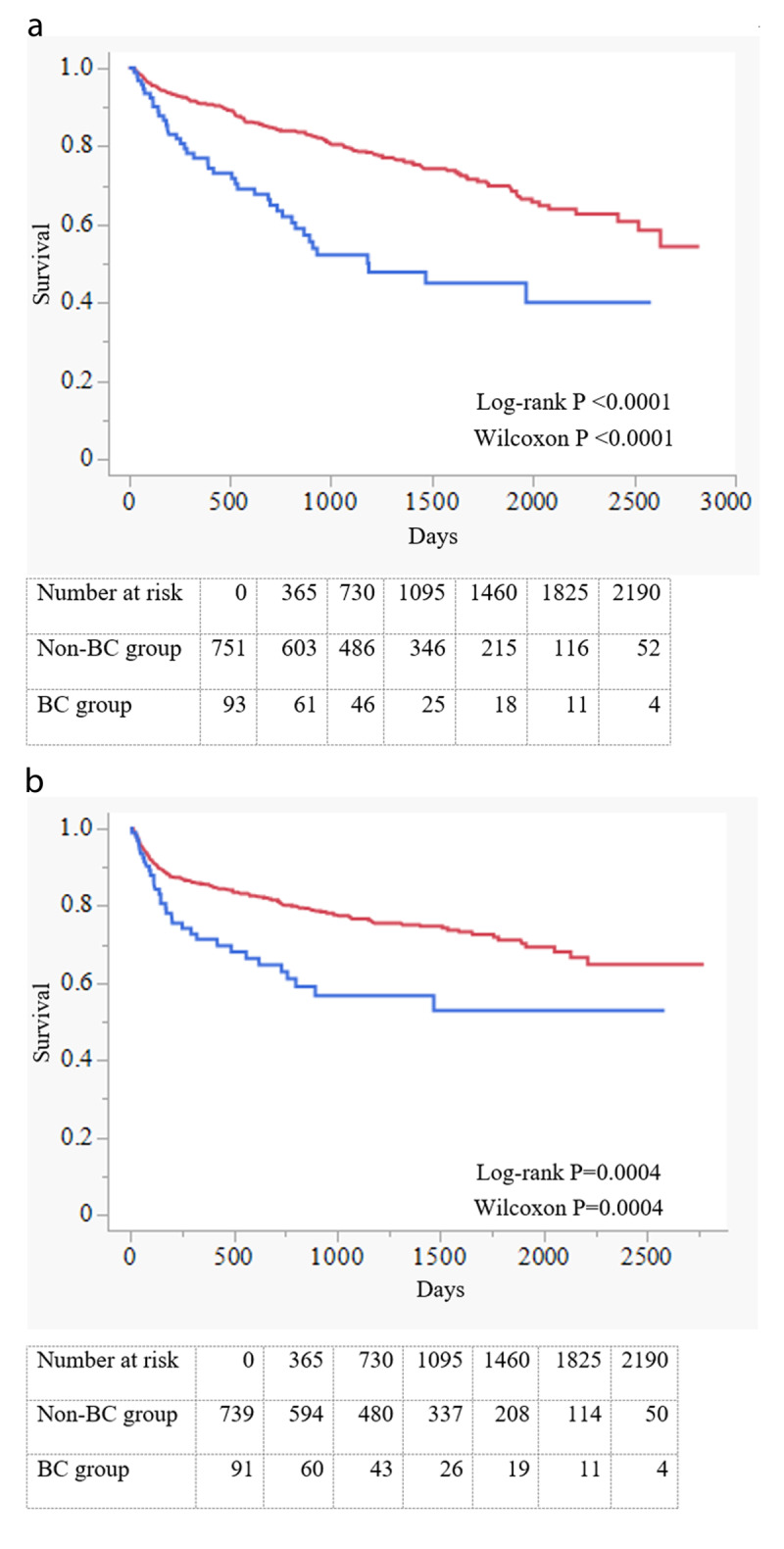
**(a)** Kaplan–Meier curves of all-cause mortality, **(b)** Kaplan–Meier curves of the time to first event (cardiovascular mortality and hospitalisation for HF).

**Figure 2:**
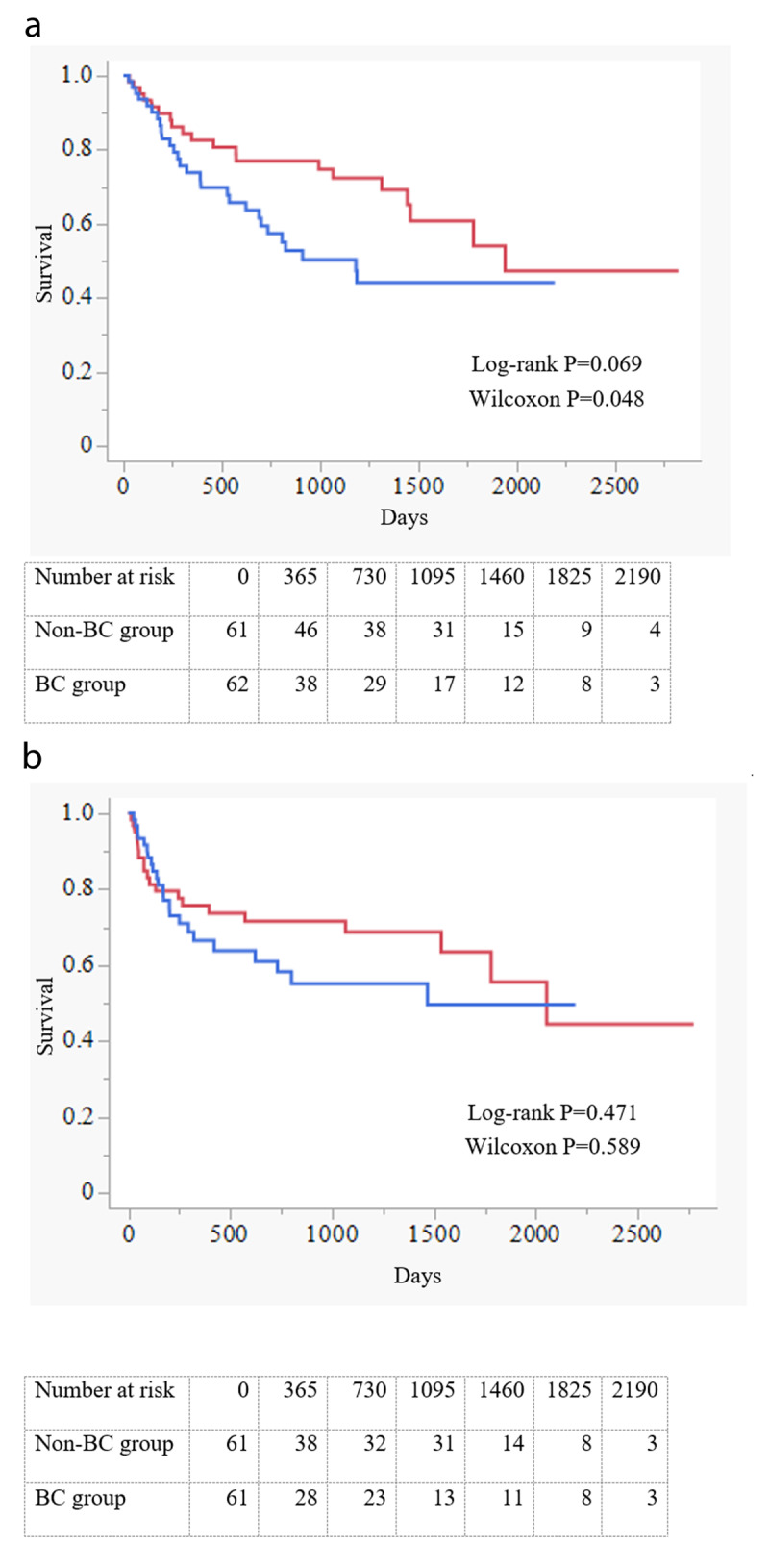
**(a)** Kaplan–Meier curves of all-cause mortality after propensity score matching, (b) Kaplan–Meier curves of the time to first event (cardiovascular mortality and hospitalisation for HF) after propensity score matching.

Among 935 hospitalised patients with HF who met the inclusion criteria from January 1, 2011 to December 31, 2020, the BC and non-BC groups included 103 and 832 patients, respectively. The baseline characteristics of the overall population are presented in Table 1. Patients in the BC group were older (mean ± SD age, 80.0 ± 12.0 vs. 73.4 ± 14.7 years; *P* < 0.0001), included a higher proportion of females (55.3% vs. 41.7%; *P* = 0.0009), and had a higher prevalence of atrial fibrillation (51.5% vs. 41.1%; *P* = 0.047). β-Blockers and ACEIs/ARBs were used significantly less frequently by patients in the BC group than by those in the non-BC group. (60.2% vs. 73.6%; *P* = 0.005 and 56.3% vs. 72.4%; *P* = 0.001, respectively). BC group patients showed a lower baseline eGFR and a higher LVEF relative to the non-BC group patients (50.8 ± 24.1 vs. 56.2 ± 23.9 mL/min/1.73 m^2^; *P* = 0.03 and 48.1 ± 16.1 vs. 42.4 ± 15.8%; *P* = 0.0008). The diameter of the inferior vena cava was larger in the BC group than in the non-BC group (18.3 ± 6.0 mm vs. 16.9 ± 4.8 mm, *P* = 0.007). The tricuspid regurgitation pressure gradient was also higher in the BC group than in the non-BC group (38.1 ± 16.3 vs. 34.3 ± 14.0 mmHg, *P* = 0.012). The median follow-up period was 951.0 days (IQR, 458.0–1513.0 days). Using unadjusted Kaplan–Meier analysis, significantly higher all-cause mortality (both of log-rank *P* and Wilcoxon *P* < 0.0001) and a composite endpoint of cardiovascular mortality and hospitalisation for HF were found in the BC group than in the non-BC group (both of log-rank *P* and Wilcoxon *P* = 0.004; Figure 1). Propensity score matching resulted in 63 matched pairs. The post-matching baseline characteristics of the BC and non-BC groups are listed in Table 2. The mean ages were 78.5 ± 11.9 years in the BC group and 76.9 ± 10.1 years in the non-BC group; 44.4% and 42.9% of the participants were male patients, respectively. After matching, a few differences remained, with an SMD of >0.20 for systolic and diastolic blood pressure, heart rate, sodium, and prescription of ACEI/ARB. The results of the Kaplan–Meier analyses after propensity score matching are shown in Figure 2. Although a composite endpoint of cardiovascular mortality and hospitalisation for HF did not differ significantly between the two groups, all-cause mortality was significantly higher in the BC group than in the non-BC group (log-rank *P* = 0.069, Wilcoxon *P* = 0.048). In the Cox proportional hazard model adjusted for the propensity score, BC was associated with higher all-cause but not composite endpoint (HR, 1.931; 95% CI, 1.251–2.982; *P* = 0.003 and HR, 1.308; 95% CI, 0.818–2.093; *P* = 0.263; Table 3). The most common cause of non-cardiovascular death was infection (40%), followed by bleeding (20%) and cancer (6%).

**Table 1:** Patient characteristics at discharge.


	NONE-BC	BC	*P*-VALUE

	*n* = 832	*n* = 103	

Male, *n* (%)	485 (58.3)	46 (44.7)	0.009

NYHA2/3/4	96/329/407	15/41/47	0.65

Age, years old	73.4 ± 14.7	80.0 ± 12.0	<0.0001

History of hospitalisation for HF, *n* (%)	149 (17.9)	32 (31.1)	0.003

Atrial fibrillation, *n* (%)	342 (41.1)	53 (51.5)	0.047

Myocardial infarction, *n* (%)	171 (20.6)	18 (17.5)	0.46

Hypertension, *n* (%)	542 (65.1)	67 (65.1)	0.98

Diabetes mellitus, *n* (%)	250 (30.1)	25 (24.3)	0.22

Hyperlipidaemia, *n* (%)	283 (34.0)	31 (30.1)	0.42

Systolic blood pressure, mmHg	113.0 ± 17.6	114.8 ± 18.9	0.43

Diastolic blood pressure, mmHg	65.6 ± 12.2	62.3 ± 12.2	0.049

Heart rate, bpm	76.6 ± 16.0	73.1 ± 14.5	0.10

Creatinine, mg/dL	1.10 ± 0.68	1.13 ± 0.57	0.70

eGFR, mL/min/1.73 m^2^	56.2 ± 23.9	50.8 ± 24.1	0.03

Sodium, mEq/L	138.4 ± 3.7	138.2 ± 4.4	0.78

BNP, pg/mL	411.5 ± 448.6	464.1 ± 413.5	0.33

White blood cell,/μL	6275.0 ± 1926.3	3933.7 ± 1628.8	<0.0001

Haemoglobin, g/dL	12.6 ± 2.4	10.2 ± 1.2	<0.0001

Platelet, 104/dL	23.3 ± 8.8	15.5 ± 7.1	<0.0001

LVEF, %	42.4 ± 15.8	48.1 ± 16.1	0.0008

HF preserved EF, *n* (%)	276 (33.1)	51 (49.5)	0.001

Medication at discharge			

*β*-Blockers, *n* (%)	612 (73.6)	62 (60.2)	0.005

ACEI/ARBs, *n* (%)	602 (72.4)	58 (56.3)	0.001

Mineralocorticoid receptor antagonists, *n* (%)	515 (61.9)	62 (60.2)	0.74

Loop diuretics, *n* (%)	556 (66.8)	79 (77.7)	0.04


ACEI, angiotensin-coenzyme inhibitors; ARB, angiotensin receptor blockers; BNP, B-type natriuretic peptide; EF, ejection fraction; GFR, estimated glomerular filtration rate; HF, heart failure; LV, left ventricular; NYHA, New York Heart Association.

**Table 2:** Patient characteristics after propensity score matching.


	NONE-BC GROUP	BC GROUP	*P*-VALUE	SD

	*n* = 63	*n* = 63		

Age, years old	76.9 ± 10.1	78.5 ± 11.9	0.439	0.1383

Male, *n* (%)	27 (42.9)	28 (44.4)	0.857	0.032

NYHA2/3/4, *n*	6/22/35	7/28/28	0.454	0.052

History of hospitalisation for HF, *n* (%)	21 (33.3)	20 (31.8)	0.849	0.034

Atrial fibrillation, *n* (%)	32 (50.8)	32 (50.8)	1.00	0

Myocardial infarction, *n* (%)	12 (19.1)	11 (17.5)	0.818	0.041

Hypertension, *n* (%)	47 (74.6)	38 (60.3)	0.086	0.310

Diabetes mellitus, *n* (%)	17 (27.0)	16 (25.4)	0.839	0.036

Hyperlipidaemia, *n* (%)	27 (42.9)	17 (27.0)	0.061	0.338

Systolic blood pressure, mmHg	117.0 ± 19.1	112.3 ± 20.3	0.332	0.236

Diastolic blood pressure, mmHg	64.7 ± 11.4	61.4 ± 12.2	0.254	0.277

Heart rate, bpm	78.6 ± 14.6	70.2 ± 10.4	0.008	0.239

Creatinine, mg/dL	1.26 ± 0.88	1.20 ± 0.60	0.646	0.082

eGFR, mL/min/1.73 m^2^	48.2 ± 22.1	47.8 ± 23.4	0.928	0.016

Sodium, mEq/L	139.2 ± 3.8	138.0 ± 3.9	0.083	0.311

BNP, pg/mL	498.9 ± 546.0	491.2 ± 435.7	0.931	0.016

White blood cell,/μL	6155.4 ± 2250.6	3995.1 ± 1813.1	<0.0001	1.057

Haemoglobin, g/dL	10.3 ± 1.4	10.3 ± 1.1	0.783	0.049

Platelet, 104/μL	23.5 ± 11.5	15.5 ± 7.1	<0.0001	0.829

LVEF, %	46.8 ± 16.4	46.7 ± 15.6	0.978	0.005

HF preserved EF, *n* (%)	34 (54.0)	34 (54.0)	1	0

Medication at discharge				

*β*-Blockers, *n* (%)	47 (74.6)	44 (69.8)	0.55	0.106

ACEI/ARBs, *n* (%)	45 (71.4)	36 (57.1)	0.093	0.302

Mineralocorticoid receptor antagonists, *n* (%)	42 (66.7)	37 (58.7)	0.357	0.165

Loop diuretics, *n* (%)	47 (74.6)	50 (79.4)	0.525	0.114


The propensity matching included age, sex, atrial fibrillation, history of hospitalization for HF, haemoglobin levels at discharge, eGFR at discharge, BNP levels at discharge, LVEF, and the prescription of *β*-blockers and angiotensin-coenzyme inhibitors/angiotensin receptor blockers at discharge. ACE, angiotensin-coenzyme inhibitors; ARB, angiotensin receptor blockers; BNP, B-type natriuretic peptide; EF, ejection fraction; GFR, glomerular filtration rate; HF, heart failure; LV, left ventricular: NYHA, New York Heart Association.

**Table 3:** The Cox proportional-hazards model of bicytopenia for all-cause mortality and composite endpoint of cardiovascular mortality and hospitalization of HF (adjusted propensity score).


	HAZARD RATIO	95% CONFIDENCE INTERVAL		*P*-VALUE

All-cause mortality	1.931	1.251	2.982	0.003

Composite endpoint	1.308	0.818	2.093	0.263


The results using univariate and multivariate Cox hazard models without propensity score matching are presented in Table 4. BC was associated with increased all-cause mortality using the multivariate Cox hazard model (HR, 1.983; 95% CI, 1.008–3.898; *P* = 0.047).

**Table 4:** Univariate and multivariate cox proportional hazard models for all-cause mortality.


	UNIVARIATE COX PROPORTIONAL HAZARD ANALYSIS				MULTIVARIATE COX PROPORTIONAL HAZARD ANALYSIS			
	
	HR	95%CI		*P*	HR	95%CI		*P*

Bicytopenia	2.523	1.791	3.553	<0.0001	1.983	1.008	3.898	0.047

Male	1.008	0.766	1.326	0.957	1.668	1.022	2.725	0.041

NYHA 3 (reference 2)	0.949	0.597	1.509	0.825				

NYHA 4 (reference 2)	1.143	0.729	1.793	0.56				

Age	1.067	1.053	1.083	<0.0001	1.069	1.041	1.097	<0.0001

History of hospitalisation for HF	1.889	1.402	2.544	<0.0001	0.927	0.527	1.629	0.792

Atrial fibrillation	1.226	0.935	1.609	0.14				

Myocardial infarction	1.661	1.234	2.237	0.001	1.306	0.764	2.232	0.33

Hypertension	1.01	0.76	1.341	0.947				

Diabetes mellitus	1.036	0.775	1.384	0.811				

Hyperlipidaemia	1.09	0.827	1.438	0.541				

Systolic blood pressure	0.999	0.988	1.009	0.86				

Diastolic blood pressure	0.965	0.948	0.982	<0.0001	0.988	0.971	1.006	0.185

Heart rate	1.005	0.992	1.017	0.451				

Creatinine	1.256	1.08	1.428	0.004				

eGFR	0.986	0.979	0.992	<0.0001	0.999	0.986	1.001	0.824

Sodium	0.969	0.935	1.007	0.104				

BNP	1.0003	1.00005	1.0006	0.022	1	0.999	1.001	0.085

White blood cell μ/L	0.999	0.999	1	0.802				

Haemoglobin	0.783	0.734	0.833	<0.0001				

Platelet	0.985	0.968	1.002	0.09				

LVEF	1.011	1.002	1.019	0.015	0.992	0.975	1.009	0.366

HF preserved EF	1.287	0.972	1.704	0.081				

*β*-Blockers	0.584	0.44	0.774	0.0002	1.07	0.554	2.064	0.841

ACEI/ARBs	0.631	0.476	0.837	0.0014	1.045	0.591	1.846	0.88

Mineralocorticoid receptor antagonists	0.722	0.55	0.947	0.019	0.731	0.454	1.177	0.197

Loop diuretics	1.191	0.882	1.608	0.254				


The variables fitted in the multivariate analysis included age, sex, history of HF admission, prior myocardial infarction, diabetes mellitus, left ventricular ejection fraction (LVEF), serum creatinine, estimated glomerular filtration rate (eGFR), BNP at discharge, and a prescription of *β*-blockers, angiotensin-coenzyme inhibitors (ACEI)/angiotensin receptor blockers (ARB) and mineralocorticoid receptor antagonist at discharge. ACEI, angiotensin-coenzyme inhibitors; ARB, angiotensin receptor blockers; BNP, B-type natriuretic peptide; EF, ejection fraction; GFR, estimated glomerular filtration rate; HF, heart failure; LV, left ventricular; NYHA, New York Heart Association.

## Discussion

In this study, the prevalence of BC in patients with HF was 11.0% (103/935). The prevalence of BC in the general population is unknown because no large-scale research has been conducted on BC. Thus, our findings suggest that blood disorders are common in patients with HF. The patients with HF and BC were older and more often female, with a higher prevalence of atrial fibrillation and higher LVEF, than were patients without BC. These characteristics are similar to those in patients with HF with preserved EF; in fact, the prevalence of HF with preserved EF was higher in patients with HF and BC.

From our findings, BC was associated with higher all-cause mortality but not cardiovascular mortality. The majority of non-cardiovascular deaths were attributed to infection, cancer, and haemorrhage. We did not perform genetic testing or bone marrow biopsy. However, myelodysplastic syndrome associated with genetic mutation and clonal haematopoiesis are common in patients with cytopenias (14). Coombs et al reported that clonal haematopoiesis is common in patients with non-haematologic cancer (15). Thrombocytopaenia and anaemia are risk factors of bleeding (16,17). Clonal haemopoiesis increased the risk of infection (18). The lack of an observed association among cardiovascular mortality, hospitalisation for HF, and BC may be attributable to the decreased sample size caused by propensity score matching. Anaemia, a condition within the spectrum of cytopenia, did show an association between HF hospitalisation and mortality in patients with chronic HF (19). In HF with preserved EF, subanalysis in the Treatment of Preserved Cardiac Function with an Aldosterone Antagonist (TOPCAT) trial showed that anaemia was associated with increased death due to malignancy. Patients with HF and anaemia in the TOPCAT trial might have overlapped with those with BC in the present study. Of note, Mentz et al. reported that anaemia at discharge after hospitalisation for HF was associated with all-cause mortality but not with cardiovascular mortality (20).

Whereas previous studies have reported that anaemia is a risk factor for mortality in patients with HF (6), the present study focussed on BC, resulting in different findings. The mechanism of BC may be distinct from that of anaemia, which could explain the observed discrepancies. Many patients with BC in the present study had anaemia (102/103).

In general, anaemia was more prevalent than BC in patients with HF. Nanas et al. reported iron deficiency as the primary mechanism of anaemia in patients with HF, with a probability of approximately 70%, followed by chronic inflammation on bone marrow biopsy (21). According to previous literature, some mechanisms underlying BC in patients with HF are speculated. The aetiologies of pancytopenia and BC are broadly categorised as production or activated destruction disorders. Production disorders are primarily caused by malnutrition or bone marrow failure. Peripheral destruction is associated with autoimmune disease and splenic sequestration (22).

Malnutrition, which includes copper and vitamin B12 deficiencies, is one mechanism of pancytopenia (23,24). A previous meta-analysis showed that 46% of patients with HF are malnourished (25). Thus, malnutrition may play an important role in the occurrence of BC in patients with HF. Another cause of BC in patients with HF is decompensated liver cirrhosis. However, hypersplenism secondary to liver cirrhosis typically leads to pancytopenia, which is not a common cause of BC (22). However, the patient data in the present study did not include the history of alcohol consumption. Persistent congestion of the liver due to HF may lead to cirrhosis (26). In the present study, the inferior vena cava diameters were similar in patients with and without BC. Although the tricuspid regurgitation pressure gradient was higher in patients with BC, whether this difference could affect liver congestion is uncertain. Since the cardiac ultrasound was performed only once during hospitalisation, the persistence of congestion after discharge could not be assessed.

An experimental study reported an association between HF and decreased haematopoietic progenitor cells via an increased apoptosis rate (27). The bone marrow progenitor cells express adrenergic receptors, suggesting the regulatory role of catecholamines in haematopoiesis (19). Sympathetic nerve activity in patients with HF might affect this decrease in haematopoietic progenitor cells (28). In patients with HF, BC occurrence is unlikely attributed to a single cause but rather to a combination of factors. Therefore, further research on the association between congestion status and blood disorders is required. Chronic inflammation occurs in patients with HF; it can be one of the treatment targets for HF. According to the site of experimental research, activation of the sympathetic nerve system increases inflammatory cells derived from bone marrow (29).

Accumulating evidence indicates that clonal haematopoiesis (as reflected by BC) is associated with worse HF outcomes (30,31). For example, patients with HF harbouring clonal haematopoiesis have significantly higher rates of death or rehospitalization over a 4-year period, independent of conventional prognostic markers, such as LVEF or N-terminal-proBNP (30). Thus, BC can be considered a potential prognostic factor and may be leveraged for risk stratification in patients with HF.

Measures to improve HF outcomes in patients with BC should be formulated. First, optimising nutritional status is one approach, as adequate nutrition may help counteract HF-related cachexia and support immune health. Second, suppression of chronic inflammation is particularly promising for those who have BC due to clonal haematopoiesis with a heightened inflammatory state. In fact, anti-inflammatory therapy has shown potential in this context: in a sub-analysis of the Canakinumab Anti-Inflammatory Thrombosis Outcomes Study (CANTOS), patients with clonal haematopoiesis exhibited increased inflammatory markers, and those with TET2-mutant clones experienced a markedly reduced rate of cardiovascular events (~60% relative risk reduction) when treated with the interleukin (IL)–1β inhibitor canakinumab (32). This finding suggests that targeting the IL-1/IL-6 pathway could improve HF outcomes in patients with BC and a heightened inflammatory state.

Based on our findings, interventions for preventing infections might reduce the risk of non-cardiovascular mortality. The strong recommendation of vaccination or rehabilitation of oral frailty for patients with BC and HF might reduce the risk of infection. Careful screening and follow-up for cancer might also reduce cancer-related mortality. Further large-scale investigations are needed to determine the mechanisms of BC in patients with HF and evaluate its relationship with prognosis.

Some limitations of the study should be acknowledged. This study employed a single-centre retrospective design, which may limit generalisability. However, we mitigated this limitation by using propensity score matching and multivariable analyses to control for key confounders (e.g., age and comorbidities), thereby strengthening the inference that BC contributes to HF outcomes. The history of cancer and blood disorders was not investigated, although it might have influenced the findings. However, patients with HF and cancer or blood disorders were admitted to specialised departments for cancer and blood disorders, where cardiologists provided consultations as needed. To minimise the influence of acute infections, we used laboratory data at discharge in the analysis. However, infections, particularly chronic infections, might not have been completely excluded. In addition, patients who died during hospitalisation were excluded; they were likely to have been in a more severe condition of HF. We also did not measure microelement and vitamin levels and did not perform the right heart catheterisation, biopsy, or bone marrow aspiration. Consequently, the analysis might have been biased towards patients with relatively lower mortality. Sodium-glucose co-transporter 2 inhibitor and angiotensin-neprilysin inhibitor were approved in August and November 2020, respectively; therefore, this might not have had a significant effect. Considering this study was conducted in a single Japanese centre, our findings may not apply to other regions. Anaemia, leukopaenia, and thrombocytopaenia were defined according to the WHO definitions, although their appropriateness in patients with HF is uncertain; however, previous studies have used these definitions. We also did not measure microelement and vitamin levels and did not perform the right heart catheterisation, biopsy, or bone marrow aspiration.

## Conclusion

BC was an important risk factor for all-cause mortality in patients with HF. Future studies should focus on the underlying pathophysiology of BC in patients with HF to identify novel therapeutic targets.

## Data Accessibility Statements

The data underlying this article will be shared on reasonable request to the corresponding author.
